# P-1088. Evaluating the Impact of Methicillin-Resistant Staphylococcus aureus (MRSA) Infections on Hospital Outcomes in Northern Jordan: A Retrospective Cohort Study

**DOI:** 10.1093/ofid/ofaf695.1283

**Published:** 2026-01-11

**Authors:** Milad Rawashdeh, Mohammad Alrawashdeh

**Affiliations:** Jordan University of Science and Technology, Irbid, Irbid, Jordan; Jordan University of Science and Technology, Irbid, Irbid, Jordan

## Abstract

**Background:**

Methicillin-resistant Staphylococcus aureus (MRSA) is a Gram-positive coccus that causes various severe infections such as skin infections and pneumonia. Despite the increase in its prevalence over the last two decades, there is a shortage of studies that evaluate the outcomes related to MRSA.

**Methods:**

We used a retrospective cohort study design to analyze electronic health records data from a large tertiary and teaching hospital between January 2018 and March 2023. Only adult inpatients with Staphylococcus aureus positive cultures were included. Antibiotic sensitivity test was used to determine the MRSA status. Logistic and negative-binomial regression models were implemented to evaluate the impact of MRSA status on patient outcomes (in-hospital death, hospital length of stay [LOS], and ICU admission) controlling for age and gender.

**Results:**

Out of 1,122 hospitalizations included in the study, 665 (59.3%) were female, and the mean age was 54.9 (SD=19.1) years. A total of 376 (33.5%) hospitalizations were admitted to the ICU, 233 (20.8%) died in the hospital, and the mean LOS was 17.2 (SD=27.0) days. Approximately three-quarters of the sample (n=837, 74.6%) had an MRSA infection. Older age alone was a significant predictor of in-hospital death (odds ratio [OR]= 1.04, 95% confidence interval [CI]= 1.03-1.05, p-value< .001). Both older age (OR=1.02, 95% CI=1.01-1.03, p-value=.003) and male gender (OR=1.35, 95% CI=1.04-1.75, p-value=.02) were positively associated with ICU admission. However, positive MRSA infection was the only factor associated with longer hospital LOS (incidence rate ratio (IRR)= 1.50, 95% CI= 1.29-1.74, p-value< .001).

**Conclusion:**

MRSA infections are widely prevalent and are associated with prolonged hospital LOS but had no significant impact on in-hospital death or ICU admission rates, which underscores the importance of targeted interventions by addressing the factors contributing to extended hospitalization.

**Disclosures:**

All Authors: No reported disclosures

